# Cystic lesions and their role in pancreatic cancer risk stratification

**DOI:** 10.1016/j.tranon.2026.102704

**Published:** 2026-02-21

**Authors:** Rebecca Lyons, Stephen G. Maher, Joanne Lysaght

**Affiliations:** a Cancer Immunology and Immunotherapy Group, Department of Surgery, School of Medicine, Trinity Translational Medicine Institute and Trinity St. James’s Cancer Institute, St. James’s Hospital, Trinity College Dublin, Dublin 8, Ireland; b Cancer Chemoradiation Research Group, Department of Surgery, School of Medicine, Trinity Translational Medicine Institute and Trinity St. James’s Cancer Institute, St. James’s Hospital, Trinity College Dublin, Dublin 8, Ireland

**Keywords:** Pre-malignant pancreatic cystic lesions, Pancreatic cancer, Risk stratification, Early intervention

## Abstract

The prognosis for patients diagnosed with pancreatic cancer has changed little over the past 4 decades. Fewer than 20 % of patients are diagnosed at a stage amenable to potentially curative surgery and therapeutic resistance remains widespread, compounded by a lack of therapeutic targets. Early detection of pancreatic cancer is notoriously difficult, due to non-specific symptoms that delay early diagnosis in addition to limited sensitivity of current imaging modalities. However, pancreatic cystic lesions (PCLs), such as intraductal papillary mucinous neoplasms (IPMNs), provide a unique opportunity for earlier disease intervention. Indeed, PCLs can be stratified by risk of malignant transformation, but current stratification guidelines remain highly contended within the field. Importantly, accumulating evidence suggests that inflammatory and immunogenetic mechanisms may influence both cyst development and malignant progression, yet these immunobiological factors are not currently integrated into PCL risk-stratification and management frameworks. In this review, we focus on the immunobiological dimension of PCLs, highlighting the interplay between chronic inflammation, immune dysregulation, and genetic alterations that may drive cystogenesis and malignant transformation. Furthermore, we assess the evidence to support integrating an immunobiological aspect to existing risk stratification guidelines to enhance identification of high-risk pre-malignant PCLs. Such integration may ultimately identify high-risk patients more accurately and inform surveillance and therapeutic intervention strategies to prevent late-stage pancreatic cancer.

## Highlights

• PC is difficult to detect and to diagnose and is frequently diagnosed at advanced stages where treatment options and efficacy are limited.
• A number of risk-factors are associated with the biological establishment of pancreatic cancer including pre-malignant pancreatic cystic lesions.
• Certain pancreatic cystic lesions possess the ability to undergo malignant transformation and are regarded as precursor lesions for pancreatic cancer.
• Risk-stratification guidelines for the diagnosis and management of pancreatic cystic lesions remains highly contended within the field.
• Incorporation of an immunobiological component to current risk-stratification guidelines of pancreatic cystic lesions may permit more accurate identification of at-risk patients for pancreatic cancer.

## Graphical abstract

**Figure:**
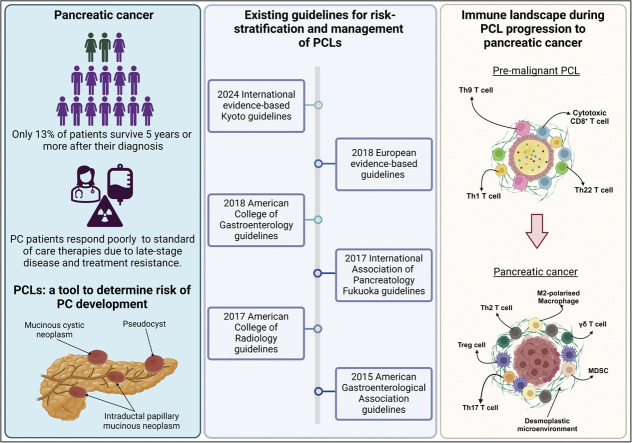

## **Introduction**

Pancreatic cancer (PC) is the third leading cause of cancer-related mortality globally, with incidence rates increasing annually [1]. Pancreatic ductal adenocarcinoma (PDAC) is the predominant subtype of PC, representing up to 95 % of all PC cases. PDAC is regarded as one of the most lethal malignant diseases in the world, with a 5-year survival rate for all PC stages combined at a dismal 13 % [1]. PC is notoriously difficult to detect and to diagnose at early stages of disease due to the aggressive nature of the cancer and late stage of clinical presentation. The symptoms associated with PC are often vague and non-specific, such as fatigue, indigestion, loss of appetite, unintended weight loss, back pain, and nausea, with early stages of disease developing asymptomatically [2]. Frequent clinical presentation of locally advanced or incurable metastatic disease occurs in up to 80 % of patients and is largely responsible for the poor 5-year survival rates in PC, which further highlights the importance of early detection and diagnosis [3]. Up to 80.9 % of patients diagnosed with PC, particularly late-stage disease, die within one year of diagnosis [4]. As a result of late-stage diagnosis, current treatment strategies primarily focus on improving quality of life and managing symptoms, as opposed to having curative intent.

**Fig. 1:**
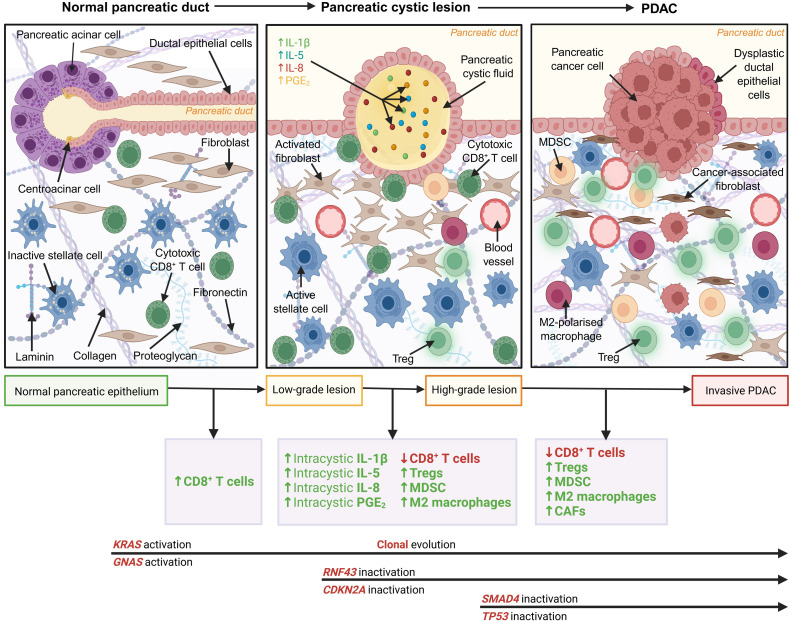
Development of a desmoplastic microenvironment from normal ductal epithelial tissue to pancreatic cystic lesion to pancreatic ductal adenocarcinoma tissue. Immune cell infiltration and therapeutic drug delivery is hindered by the dense desmoplastic microenvironment. With cancer progression, marked by increased dysplasia of ductal epithelial cells, ECM components and α-smooth muscle actin fibroblasts become more abundant. PCLs frequently accumulate genetic mutations in *KRAS, CDKN2A, TP53, SMAD4, GNAS*, and *RNF43* with progression of disease. Immune cell infiltration is reported to evolve from increased cytotoxic CD8^+^ T cells in low-grade dysplastic PCLs to a more immunosuppressive microenvironment in high-grade PCLs and invasive PDAC governed by Tregs, myeloid-derived suppressor cells (MDSCs) and M2-polarised macrophages. Tumour cells secrete growth and survival factors which cause cancer-associated fibroblasts (CAFs) and T cell (Tregs) to produce immunosuppressive cytokines and subsequently promote the proliferation, invasion, migration, and metastatic characteristics of pancreatic stellate cells and PC. ECM components, including collagen, fibronectin and laminin, are upregulated during progression of disease, ultimately perpetuating a dense desmoplastic stromal environment. Figure created with BioRender.com.

A contributing factor to the poor outcomes for patients with PC is the lack of immune cell infiltrate, termed an “immunologically cold” tumour. “Immunologically cold” tumours are typically characterised by poor CD8^+^ T cell infiltration into the tumour microenvironment (TME). Additionally, these “immunologically cold” tumours often harbour immunosuppressive immune cell populations, such as regulatory T lymphocytes (Tregs), myeloid-derived suppressor cells (MDSCs), and tumour-associated macrophages (TAMs), which ultimately function to dampen the anti-tumour immune response [5,6]. Conversely, “immunologically hot” tumours are characterised by high levels of CD8^+^ T cell infiltration, increased programmed cell death ligand 1 (PD-L1) expression and high tumour mutational burden (TMB), features that are also associated with enhanced response to immune checkpoint blockade [5,6]. Extensive desmoplasia is a prominent pathological feature of the PC tumour microenvironment [7] (Fig. 1). Desmoplasia consists of a dense extracellular matrix (ECM) and a high level of α-smooth muscle actin (α-SMA)-positive fibroblasts [8]. Tumour cells are known to secrete growth and survival factors, which ultimately result in the proliferation, differentiation and activation of cancer-associated fibroblasts (CAFs) [[9], [10], [11]]. TGF-β1 is one such growth factor expressed by tumour cells that leads to ECM deposition and tissue fibrosis [12]. Consequently, activated CAFs secrete immunosuppressive cytokines and chemokines, such as IL-1β, IL-6, CXCL1, CXCL2, CXCL12, CXCL13, CCL-5, and GM-CSF, in addition to ECM components including collagen, fibronectin, laminin and matrix metalloproteinases, which promote the proliferation, invasion, migration and metastatic characteristics of PDAC cells and pancreatic stellate cells [13]. Activated CAFs promote fibrosis via collagen deposition and ECM modulation, which ultimately perpetuates the development of the desmoplastic stroma [14]. This desmoplastic stroma acts as a physical barrier preventing adequate vascularisation, hindering anti-tumour immune cell infiltration, and resistance to systemic treatments [14].

PC is widely classified as an “immunologically cold” tumour. The immunosuppressive desmoplastic microenvironment in the pancreas is heavily induced and facilitated by Tregs, MDSCs, and anti-inflammatory M2 macrophages. In an *in vivo* study carried out by Clark *et al.*, Tregs, MDSCs, and macrophages were shown to dominate the immune infiltration in pre-malignant pancreatic cystic lesions (PCLs) and persist to invasive PC [15]. PC cells secrete immunosuppressive, pro-tumorigenic cytokines including IL-6, IL-8, IL-10, IL-13, TGF-β, and VEGF, in addition to chemokines, such as CXCL12, into the TME[16]. The abundant secretion of these immune factors into the tumour microenvironment induces immune suppression by activating and recruiting Tregs, Th2 cells, MDSCs, and M2 macrophages [16,17]. These immune cell subsets block the anti-tumour activity of cytotoxic CD8^+^ T cells, effector CD4^+^ T cells, and NK cells [17]. Significantly increased populations of Tregs have been identified in the peripheral blood and stromal areas of pancreatic tissue from PDAC patients [18]. A study conducted by Hiraoka *et al.* examined clinical samples of precursor lesions and demonstrated that the accumulation of Treg cells and diminished cytotoxic CD8^+^ T cell infiltration correlated with the malignant progression of pre-neoplastic lesions [19]. These results were corroborated by additional independent studies [20,21]. However, recent multiplex immunofluorescence studies investigating the immune microenvironment of resected tissue from precursor lesions of PC found that there was increased CD8^+^ *T* cell infiltration in pre-malignant lesions [22,23].

The hostile desmoplastic microenvironment also plays a prominent role as a barrier to treatment and ultimately contributes to poor prognosis for PC patients. Since fewer than 20 % of newly diagnosed PC patients are considered eligible for surgery, identifying patients at earlier stages of PC development is imperative to improved prognosis and survival [24].

Incidence and mortality rates of PC have remained stable or slightly increased across many countries, likely reflective of the increasing prevalence of obesity, smoking, and alcohol consumption, which are classified as modifiable risk factors of PC [25,26]. Age, gender, genetically driven risk factors, such as von Hippel Lindau (VHL) disease and inflammation-driven risk factors, including diabetes and pancreatitis also incur non-modifiable risk of PC development [27]. A notable risk factor of PC development and progression is pre-malignant PCLs, which are often considered precursor lesions of PC [28,29].

### Potential avenues for enhanced risk stratification and earlier PC diagnosis

PCLs are localised fluid-filled structures that can be found within or on the surface of the pancreas. The vast majority of PCLs are detected incidentally during routine investigations, giving them their pseudonym “incidentalomas” [30,31]. While many pancreatic lesions are benign, others such as pancreatic intraepithelial neoplasia (PanIN), intraductal papillary mucinous neoplasms (IPMNs) or mucinous cystic neoplasms (MCNs), possess the ability to undergo malignant transformation and can be regarded as precursor lesions of PC [28,29,32]. Understanding the mechanisms involved in cystogenesis and tumorigenesis may be the key to identifying avenues of earlier PC diagnosis and intervention.

Non-neoplastic cysts are benign lesions that can be further classified into epithelial or non-epithelial subtypes [33]. Non-neoplastic cysts account for up to 6.3 % of all resected cysts and include pseudocysts, retention cysts, benign epithelial cysts, lymphoepithelial cysts, squamous lined cysts, mucinous non-neoplastic cysts, and lymphangiomas [33].

Pseudocysts are generally considered the most common subtype of non-neoplastic cysts [34]. Pseudocysts predominantly develop as a complication of alcohol-induced, biliary or traumatic acute pancreatitis (AP) [35]. Pseudocysts are not classified as true cysts given their presentation as amylase-rich fluid containing cellular debris, blood, and inflammatory cells encased in a fibrous wall without an epithelial lining, as would typically be found in other cysts [36,37]. Pancreatic pseudocysts also have been shown to contain inflammatory cells, such as neutrophils and tissue-resident macrophages [38]. However, further studies are required to establish a link between the expression of specific immune cells and factors in neoplastic PCLs and the risk of malignant transformation.

Neoplastic cysts are broadly categorised as serous, as in the case of serous cystic neoplasms, or mucinous cysts such as IPMNs, and MCNs [39]. Serous cystic neoplasms are generally classified as benign, whereas mucinous neoplasms are considered to have a malignant potential [33]. There are a number of biomarkers currently used in clinical practice to differentiate mucinous from non-mucinous pancreatic cysts [40,41]. Among these, intra-cystic carcinoembryonic antigen (CEA) is one of the most extensively studied biomarkers for the diagnosis of neoplastic mucinous cysts [40,41]. A CEA concentration greater than 192 ng/mL demonstrates a sensitivity of 50–75 %, specificity of 84–92 %, and an overall diagnostic accuracy of approximately 79 % for distinguishing mucinous from non-mucinous cysts [[41], [42], [43]]. In parallel, a recent meta-analysis of pancreatic cyst fluid biomarkers reported that glucose concentrations below 50 mg/dL accurately identified IPMNs and MCNs with 93 % sensitivity and 76 % specificity [40]. Mutational profiling of pancreatic lesions has proven valuable for distinguishing between different subtypes of precursor lesions [[44], [45], [46]]. Low-grade PanINs commonly harbour mutations in *KRAS* and *p16/CDKN2A*, whereas high-grade PanINs and invasive PC exhibit additional alterations in *TP53* and *SMAD4* [47,48]. Similarly, IPMN lesions frequently harbour mutations in *KRAS, CDKN2A, TP53, SMAD4, GNAS*, and *RNF43* [[49], [50], [51]]. Notably, next-generation sequencing (NGS) studies have demonstrated that the presence of *KRAS* or *GNAS* mutations is highly specific (98 % specificity) for identifying mucinous cysts, providing valuable molecular markers for distinguishing these lesions from non-mucinous counterparts [40,41]. In contrast, MCNs share a comparable mutational profile with IPMNs, but typically lack *GNAS* mutations [51]. It is yet to be elucidated whether genetic mutations associated with these lesions arise coincidentally and induce neoplastic changes within the cyst or otherwise arise as a consequence of exposure of cyst epithelial lining to pancreatic cyst fluid, which further perpetuates the neoplastic process. In a study conducted by Singhi *et al.*, DNA from isolated cyst epithelial lining that had shed into human pancreatic cyst fluid underwent NGS [44]. This study showed that *KRAS*/*GNAS* mutations in combination with alterations or deletions, particularly in *TP53, SMAD4, PIK3CA, p16/CDKN2A* and *PTEN* were associated with advanced neoplasia [44].

PanINs are non-cystic precursor lesions responsible for more than 90 % of diagnosed PCs [52,53]. However, despite their clinical significance, there is currently no established method for surveillance or early detection of PanINs [52,53]. These lesions are characteristically asymptomatic in nature and remain undetectable on standard radiological imaging due to their microscopic size [52,53]. Molecular alterations associated with PanINs have been shown to accumulate with increasing grades of dysplasia and eventually lead to uncontrolled cell proliferation and subsequent progression to invasive carcinoma [54]. These alterations include activating point mutations to *KRAS*, an oncogene that acts as a mediator for cell-cycle progression and proliferation [55,56]. Other molecular alterations identified in these precursor lesions include inactivation of point tumour suppressor genes *TP53, p16*, and *SMAD4* [57,58].

IPMNs account for up to 38 % of neoplastic PCLs [59]. IPMNs are comprised of mucin-producing columnar cells arising in the main duct (MD), branch duct (BD) or a combination of both types (mixed type (MT)) in the pancreas [60]. These precursor lesions develop papillary proliferation, cyst formation and varying degrees of cellular atypia [49,61]. Patients with MD-IPMNs and MT-IPMNs have been shown to be at an increased risk of malignant development, with approximately 45 % having an associated invasive carcinoma compared to BD-IPMNs, where approximately 15 % of diagnosed cases are associated with the development of invasive carcinoma [[62], [63], [64]]. In contrast to PanINs, IPMN lesions are frequently detectable on radiological imaging, which makes clinical surveillance feasible [65,66]. Their detection has significant clinical implications, as IPMNs possess malignant potential to progress to invasive PC [67,68]. A key benefit of image-based surveillance is the detection of PC at an earlier, more treatable stage. In a large retrospective study of 450 patients, cancers detected in individuals undergoing surveillance were significantly more likely to be Stage I compared to those diagnosed in patients proceeding directly to surgery (69.2 % vs. 37.3 %), providing robust, data-driven justification for PCL surveillance [69]. Furthermore, the prevalence of IPMNs in the adult population is estimated to be approximately 10–20 %, underscoring their importance as a common and clinically relevant PCL [65,70]. IPMNs are graded based on the degree of dysplasia, which can be classified as low-grade dysplasia (adenoma) and high-grade dysplasia (carcinoma in situ) [[71], [72], [73]]. Additionally, in light of their high prevalence and malignant potential, several clinical guidelines have been established to stratify the risk of progression based on the presence of specific “worrisome features” [74]. Reported estimates suggest that low-risk IPMNs carry a 0.02–7.77 % probability of developing into invasive PC, whereas high-risk IPMNs have a markedly higher risk, ranging from 14.4–47.9 % [67,68].

Initial studies investigating the hypothesis that a proinflammatory environment exists in IPMN patients have found that there was significantly elevated IL-1β levels in addition to overexpression of other inflammatory markers, such as IL-5 and IL-8, in high-risk IPMN cyst fluid in comparison to low-risk IPMN cyst fluid [75]. IL-1 is primarily synthesised by monocytic phagocytes that have underwent stimulation in immunogenic and pro-inflammatory environments [76]. IL-1β is secreted into the extracellular space as opposed to intracellular, membrane-bound IL-1α, which enables this cytokine to be measured in pancreatic cyst fluid [76]. The presence of IL-1β in these dysplastic cysts in the pancreatic setting may reflect how the inflammatory microenvironment plays a role in malignant progression. Some studies have found that elevated IL-1β expression in tumour tissue is associated with increased cancer risk and poorer survival in a multitude of other neoplasms, including ovarian and cervical cancer, and mediated PC cell invasion [[77], [78], [79]]. Cell mediated and humoral immune responses are facilitated through Th1 and Th2 CD4^+^ T lymphocytes, respectively, whereby immune activity can be measured through quantification of cytokines such as IL-1β, IL-2, IL-4, IL-5, IL-8, IL-10, IL-12, IL-13, IFN-γ, and TNF-α [80]. IL-1β signalling also assists in the activation of CD4^+^ T lymphocytes in an antigen dependent manner, whereby this process may be triggered when low-grade dysplastic IPMNs transform into highly dysplastic IPMNs [81].

**Fig. 2:**
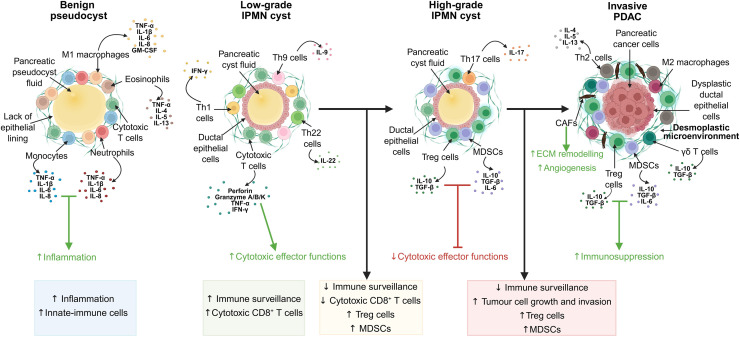
The evolution of the immune T cell landscape during progression of pancreatic IMPN cysts to the development of invasive PDAC. Benign pseudocysts are primarily governed by inflammatory innate-immune cells, such as macrophages, monocytes and neutrophils. T cell phenotypes are primarily cytotoxic and pro-inflammatory in pre-malignant lesions, such as low-grade IPMN lesions. With progression of pancreatic IPMN lesions from low-grade dysplasia to high-grade dysplasia, changes occur in the hierarchy of T cell phenotypes in the immune microenvironment as regulatory T cells, Th2 cells, Th17 cells, and γδ T cell subsets work to suppress effector T cell infiltration and, in turn, hinder immune surveillance. With increased levels of dysplasia, IPMN lesions also develop an immunosuppressive landscape through an increased infiltration of Treg cells and MDSCs which are known to suppress cytotoxic effector functions. The TME of PDAC is also predominantly composed of immunosuppressive cell types (Tregs, MDSCs, M2-polarised macrophages), but also of cancer-associated fibroblasts which contribute to extracellular matrix remodelling and increased angiogenesis. The immunosuppressive microenvironment of PDAC mediates tumour growth, immune evasion, and metastasis. Figure created with BioRender.com.

A study carried out by Roth *et al.* assessed tissue samples that were obtained from IPMN resections using immunohistochemical analysis to examine the spatial distribution of immune cell infiltrates during IPMN progression [82]. Findings from this analysis established that the immune microenvironment evolved from a diverse T cell arena, which is comprised of CD8^+^ T cells, Th/c1, Th/c2, Th/c17, Th22, and Treg cells in low-grade IPMNs to a primarily Treg dominated immunosuppressive state during the carcinogenesis of IPMNs and invasive PC [82]. This study also demonstrated the formation of tertiary lymphoid structures in the surrounding stroma of IPMNs, which accumulate immunosuppressive cell types and subsequently evolve during progression to invasive PC [82]. However, the results of these studies further emphasise the need to examine the altered immune components in cyst fluid to understand the mechanisms used by localised cells within these neoplastic cysts that ultimately promote malignant transformation (Fig. 2). Nevertheless, this emerging evidence suggests that composition of the T-cell repertoire in pre-malignant IPMN tissue may reflect changes in immune dynamics predictive of cyst progression and may have potential utility as an immunobiological biomarker for integration into PCL risk stratification and management guidelines.

MCNs are a rarer subtype of neoplastic pancreatic lesion, accounting for up to 23 % of PCLs [59]. More than 95 % of MCNs develop in the body and tail of the pancreas and histologically display characteristically tall, columnar, mucin-producing epithelial cells [83]. MCNs generally arise in the middle-aged female population and can be differentiated from IPMNs due to the ovarian stromal involvement associated with MCNs [84]. The preponderance of MCNs in females, in addition to the presence of oestrogen receptors has led to the hypothesis that as a result of ectopic ovarian stroma being incorporated during embryogenesis in the pancreas, the release of hormones and growth factors cause adjacent epithelium to proliferate and form cystic tumours [85]. There are no distinguishable factors between MCNs that can progress to invasive carcinoma and those that remain benign and so resection of these cysts is typically recommended [84].

### Comorbidities associated with increased incidence of PCLs

#### *Pancreatitis*

Inflammation of the pancreas, otherwise termed “pancreatitis”, is a significant risk factor for PC development and has been established as a critical factor in the initiation and maintenance of the pancreatic disease state [15,[86], [87], [88]]. The immunosuppressive environment cultivated by the pancreatic stroma in PC, has been hypothesised to promote tumour progression and invasion [89]. Acute pancreatitis (AP) is one of the most commonly diagnosed gastrointestinal conditions with global incidence rates increasing annually [90]. AP presents with increased serum concentrations of amylase and lipase [91] in addition to a systemic decrease of circulating T lymphocytes, B lymphocytes, and natural killer (NK) cells [[92], [93], [94]]. Recurrent bouts of AP, often instigated through exposure to risk factors such as alcohol consumption, gallstones, and smoking, leads to fibrosis and chronic pancreatitis (CP), which is an inherent risk factor for the development of PC [[95], [96], [97]]. Although CP shares many of the same risk factors as AP, the clinical presentation differs dramatically. In contrast to the elevated serum amylase and lipase levels in AP, these pancreatic enzymes are generally normal or only slightly elevated in CP [98]. CP can instead be characterised by extensive pancreatic fibrosis, acinar cell atrophy, and immune cell infiltrate [99]. Chronic inflammation of the pancreatic tissue, as in the case of CP, constitutively activates pro-inflammatory cytokines such as IL-6, IL-8, IFN-γ and TNF-α, and are found to be increased in both CP and PDAC [100,101]. The longstanding inflammation of CP is characterised by macrophage, T lymphocyte and B lymphocyte infiltration into pancreatic tissue [[102], [103], [104]], whilst the CD4^+^ and CD8^+^ T lymphocyte population appears to decrease in peripheral blood [105,106].

Uncontrolled activation of pancreatic acinar cells and release of digestive enzyme stores leads to the autodigestion of pancreatic cells [107]. Autodigestion of these cells releases cellular contents that trigger recruitment of inflammatory cells, which ultimately release cytokines and immunomodulatory factors such as IL-1β, IL-6, IL-8, IL-33, TNF-α, MCP-1, and MIP-2 [[108], [109], [110], [111]].

In response to pancreatitis-driven inflammation, pancreatic acinar cells can undergo acinar-to-ductal metaplasia (ADM), a key tumour initiation mechanism of PDAC [[112], [113], [114]]. ADM is transient and reversible in patients with pancreatitis or pancreatic injury [115]. However, the molecular mechanism driving formation and malignant progression following persistent ADM is yet to be elucidated. An *in vivo* study carried out by Liou *et al.* found that macrophage-secreted chemokines and cytokines such as RANTES, which functions as a potent chemoattractant for a multitude of immune cells including T cells, and TNF-α, promote ADM through activation of NF-κB and matrix metalloproteinases [87]. Constitutive NF-κB activation has previously been described as an early distinguishing factor of both AP and CP, in addition to PDAC cells in comparison to normal pancreatic tissue [[116], [117], [118], [119]].

#### *Diabetes mellitus*

Diabetes mellitus (DM) is a global health problem caused by chronic high glucose levels in the blood as a result of dysfunctional β cells in the pancreas, which fail to produce adequate insulin or otherwise incur ineffective insulin functionality [120]. In many patients, DM, particularly new-onset DM, often precedes the clinical diagnosis of PC [121]. However, the mechanisms underlying this association are not yet fully understood.

Some studies suggest that new-onset DM may be a predictive marker of malignant progression of pancreatic cysts, particularly so in the case of patients who develop new-onset diabetes subsequent to the development of a cystic lesion [[121], [122], [123]]. To date, only a minimal number of epidemiological studies have been published which aimed to determine the frequency of PCL incidence in DM patients and vice versa [124,125]. Although these studies fail to indicate a specific factor responsible for cyst formation in patients with DM, an increased incidence of PCLs has been associated with the aging population, those with a high body mass index (BMI) and DM history [125].

#### Von Hippel-Lindau (VHL) disease

The formation of precursor lesions in the pancreas is often influenced by genetic factors as in the case of Von Hippel-Lindau (VHL) disease. VHL disease is a hereditary autosomal dominant genetic condition resulting from deletions or germline mutations of the *VHL* gene, an established tumour suppressor gene, on chromosome 3p [126,127]. Loss of heterozygosity and somatic inactivation of the wild-type *VHL* allele have been identified within VHL-associated lesions like PCLs [128^,^^129^]. Typically, the manifestation risk of pancreatic neuroendocrine tumours associated with VHL disease is estimated at 10–17 % [130,131]. Mutations in the *VHL* gene are over 99 % specific for serous cystadenomas, and their detection often provides clinicians confidence to discontinue surveillance due to their benign nature [40]. Interestingly, up to 70 % of patients with VHL disease will develop PCLs [132,133]. However, no association has been reported between the presence of PCLs in VHL disease and malignancy [134].

A whole-exome sequencing study was performed on DNA from microdissected human neoplastic PCLs whereby the results revealed that although loss of the chromosome 3p alleles in the *VHL* gene initiates cystogenesis in pancreatic cystadenomas, IPMNs did not show any alterations in 3p alleles [50]. These lesions with malignant potential indicated that other genes encoding E3 ubiquitin ligases were involved [50]. This suggests that protein turnover may be a major contributing factor in the underlying cystogenesis mechanism.

### Biological framework for the risk-stratification and management of pre-malignant PCLs

Due to the low accuracy of image-based tests and the invasive nature of surgical biopsy, endoscopic-ultrasound fine-needle aspiration (EUS-FNA) has gained popularity as an alternative technique to evaluate PCLs. This diagnostic modality is used clinically and has been shown to have low sensitivity and accuracy (45.4 % and 57.5 % respectively) but high specificity (87.5 %) for the detection of PCLs [135]. A major limitation of this technique is the low cellularity of cyst fluid samples, which often precludes definitive cytological diagnosis [136]. To overcome these challenges, the field has evolved with the development of novel EUS-based tools that enhance tissue acquisition and real-time cyst characterisation. Through-the-needle biopsy (TTNB) employs micro-forceps passed through the EUS needle to obtain targeted tissue samples from the cyst wall or mural nodules, resulting in a significantly higher diagnostic yield compared to FNA alone [137]. Meanwhile, needle-based confocal laser endomicroscopy (nCLE) enables real-time, high-resolution microscopic imaging of the cyst epithelium, allowing for the *in vivo* differentiation of PCL subtypes based on characteristic visual identifiers [138]. In addition to diagnostic advances, EUS-guided ablation has emerged as a minimally invasive therapeutic alternative for patients who are poor surgical candidates or decline surgery [139]. This technique involves the injection of ablative agents directly into the cyst cavity to induce epithelial necrosis and cyst resolution [139]. EUS-guided ablation does not eliminate the need for ongoing surveillance, due to possible recurrence of the lesion or incomplete ablation. These advances to EUS technologies represent a substantial improvement in the endoscopic evaluation of PCLs, emphasising the need for a multimodal diagnostic approach that integrates gastroenterologist expertise with cytological, radiological, and cyst fluid analyses [140].

There are currently six major sets of guidelines presently in use to guide referrals for surveillance, EUS-FNA or surgical resection procedures for patients with clinical presentation of asymptomatic PCLs [74]. These include the 2024 International evidence-based Kyoto guidelines [141], 2018 European evidence-based guidelines (EEG) [142], the 2018 American College of Gastroenterology (ACG) guidelines [143], the 2017 International Association of Pancreatology Fukuoka guidelines [68], the 2017 American College of Radiology (ACR) guidelines [144], and the 2015 American Gastroenterological Association (AGA) guidelines [145] (Table 1).

**Table 1:** Indications for the management of PCLs based on current clinical guidelines.

**Guideline Publications**	Indications for EUS* surveillance	**Indications for surgical resection**	**Reference**
2024 International evidence-based Kyoto guidelines	*Any of the following worrisome features;* Acute pancreatitis, Elevated serum CA19-9, New onset/exacerbation of diabetes mellitus, Cyst ⩾30 mm, Thickened/enhancing cyst walls, Main PD ⩾5 mm and <10 mm, Enhancing mural nodule ⩾5mm Lymphadenopathy, Rapid growth of cyst (⩾2.5 mm/yr) during surveillance	Repeated acute pancreatitis to worsen patient’s quality of life, Multiple “worrisome features”, Cytology suspicious or positive for malignancy or HG dysplasia, Obstructive jaundice with lesion presenting in head of pancreas, Enhancing mural nodule ⩾5 mm or solid cystic component, Main PD dilation ⩾10 mm.	[141]
2018 European evidence-based guidelines (EEG)	Any clinical or radiological features of concern for malignancy, Results are expected to change clinical management.	*Relative indications:* Rapid growth of cyst (⩾5 mm/yr) during surveillance, Elevated CA–19–9 levels (37 U/mL), Main PD dilation between 5 and 9 mm/ cyst size ⩾40 mm, Acute pancreatitis/ new-onset diabetes mellitus, Enhancing mural nodule ⩾5 mm. *Absolute indications:* Cytology suspicious or positive for malignancy or HG dysplasia, Solid cystic component, main PD ⩾10 mm, Patient presenting with symptomology.	[142]
2018 American College of Gastroenterology (ACG)	*Any of the following worrisome features;* Main PD ⩾5 mm, IPMN or MCN⩾30 mm, Change in PD calibre with upstream atrophy, Rapid growth of cyst (⩾3 mm/yr) during surveillance, Jaundice, Pancreatitis, Mural nodule or solid cystic component.	IPMN or MCN ⩾30 mm and/or cyst growth (⩾3 mm/yr), Cytology with concerning features of HG dysplasia or invasive disease, Non-enhancing mural nodule/solid cystic component, New-onset diabetes, Jaundice secondary to cyst, Acute pancreatitis secondary to cyst, Elevated CA-19-9 levels, Focal dilation of main PD concerning for obstructing lesion.	[143]
2017 International Association of Pancreatology Fukuoka	*Any of the following features;* Pancreatitis due to cyst, Cyst size (⩾30 mm) with rapid growth (⩾5 mm/2 yrs) during surveillance, Thickened/enhancing cyst walls, Abrupt change in main PD diameter with distal pancreatic atrophy, Lymphadenopathy, Elevated CA-19-9 levels.	Obstructive jaundice with lesion presenting in head of pancreas, Enhanced mural nodule ⩾5 mm, Main PD dilation ⩾10 mm, Cytology suspicious or positive for HG dysplasia or invasive cancer.	[68]
2017 American College of Radiology (ACR)	Cyst size (⩾30 mm), Thickened cyst wall, Non-enhancing mural nodule, Main PD diameter ⩾7 mm.	Jaundice secondary to cyst, Enhancing solid cystic component, Main PD dilation ⩾10 mm, Cytology suspicious or positive for HG dysplasia or invasive cancer.	[144]
2015 American Gastroenterological Association (AGA)	*Two or more high-risk features;* Cyst size (⩾3 mm), Dilated main PD.	Cytology with HG dysplasia or invasive cancer, Dilated main PD, Solid cystic component.	[145]

EUS=endoscope ultrasound. PD=pancreatic duct. HG=high-grade. IPMN=intraductal papillary mucinous neoplasm. MCN=mucinous cystic neoplasm.

These stratification guidelines consider a number of clinical factors at varying degrees of priority when establishing risk of malignancy. Clinical experience of PCLs in coalition with the knowledge of current stratification guidelines, heavily influences PC risk stratification. Clinical factors considered during risk stratification include cyst size, dilation of the main pancreatic duct, presence of a mural nodules, serum carbohydrate antigen 19-9 (CA19-9) levels, diagnosis of VHL disease, development of jaundice or newly diagnosed DM [74]. These criteria and thresholds for intervention vary considerably among these guidelines. The 2015 AGA and 2017 ACR guidelines tend to adopt a more conservative approach, recommending EUS-FNA or surgical resection only when multiple high-risk features are present, thereby minimising unnecessary interventions [144,145]. In contrast, the 2017 Fukuoka, 2018 ACG and 2018 European evidence-based guidelines prioritise an extended evaluation by incorporating factors such as cyst growth rate, CA19-9 elevation, or new-onset DM as potential indications for intervention [68,142,143]. The recently updated 2024 Kyoto evidence-based guidelines expand upon this framework by emphasising long-term surveillance intervals and patient-specific risk stratification [141]. Overall, these stratification guidelines evidently exhibit a vast lack of consensus between institutions and geographical locations [68,[142], [143], [144], [145]], emphasising the need for more robust and standardised set of PCL characterisation guidelines to minimise surgical intervention and indicate for necessary surveillance.

Despite the risk of tumorigenesis associated with PCLs, particularly in the case of MCNs and IPMNs, there are no existing screening programs to detect these pre-malignant lesions [28,29]. Most PCLs are diagnosed incidentally once patients undergo routine image-based tests for other medical indications [30,146]. Accurately stratifying patients with high-risk pre-malignant PCLs will enable appropriate longitudinal surveillance to identify patients who are bona fide candidates for prophylactic surgical intervention and prevent unnecessary surgery and surveillance for patients at a lower risk of PC development. The current therapeutic landscape for PC has made minimal improvements to the 5-year survival of patients particularly due to the late-stage diagnosis, development of therapeutic resistance and a lack of druggable targets.

Immune biomarkers have shown clear clinical utility in several established cancers, such as colon cancer and breast cancer, as prognostic and sometimes predictive tools [[147], [148], [149], [150], [151]]. In colon cancer, the immunoscore assay quantifies CD3^+^and cytotoxic CD8^+^ T cell densities in tumour tissues by digital pathology [147,148]. This immune-based assay consistently outperformed or otherwise complemented traditional TNM staging in predicting recurrence-free and overall survival across large multicentre cohorts of patients with colon cancer [147,148]. Similarly, tumour-infiltrating lymphocytes (TILs) have emerged as prognostic and sometimes predictive markers in breast cancer, particularly in triple-negative and HER2-positive subtypes [149,150]. High stromal TIL counts correlate with improved disease-free survival and enhanced benefit from chemotherapy and anti-HER2 therapy [149,150]. Indeed, the expression of immune checkpoint molecule PD-L1 and tumour mutational burden (TMB) also serve as independent predictive biomarkers guiding immune-checkpoint inhibitor therapy in a number of solid malignancies [151]. These examples demonstrate that immune factors can directly inform therapeutic decisions for patients with cancer. In pre-malignant conditions, such as Barrett’s oesophagus and cervical intraepithelial neoplasia, changes in immune cell composition and cytokine expression profiles within the immune microenvironment in the early stages of the neoplastic sequence can be associated with progression risk [152,153]. Although immune biomarkers have yet to be clinically validated for use in pre-malignant conditions, their demonstrated utility in established cancers highlights their potential to improve early detection and risk stratification.

### Potential immunobiological biomarkers for cancer risk stratification of PCLs

At present, the only biomarker approved by the FDA for the detection of PC, is serum CA19-9 [154]. However, use of CA19-9 is not recommended for use in early stage PC screening approaches due to its limited specificity (75 %) and sensitivity (80 %) [155]. CA19-9 is also known to perform poorly as a biomarker in patients with inflammation-driven co-morbidities already associated with PC, such as pancreatitis, and diabetes [154]. A number of studies have emerged to overcome the issue of limited sensitivity and specificity of CA19-9 by investigating the applicability of multi-biomarker panels [156,157].

CA19-9 is not elevated in pre-malignant PCLs, emphasising the need for the addition of novel biomarkers in the pre-malignant setting to current cancer risk stratification guidelines in order to accurately classify PCLs [158]. To identify potential biomarkers that could guide early PC detection and intervention, some studies have employed liquid chromatography-tandem mass spectrometry to establish a comprehensive proteomic profile of human pancreatic cyst fluid [159,160]. Although these studies identified protein family members of mucins, CEACAMs, amylase, and SERPIN proteins, which show potential in providing a more accurate diagnosis of high-risk pre-malignant PCLs, differential expression of immune-based factors between risk-stratified PCLs was not significant [159,160]. Interestingly, Th1 and Th2 immune profiles of cytokine markers have been shown to distinguish patients with PC from patients with CP and normal pancreatic tissue in both serum and pancreatic juice samples [80,161]. It is yet to be defined whether inflammation within PCLs leads to severe dysplasia or whether increased levels of PCL-associated dysplasia initiates an immune response. Under both circumstances, identification and quantification of specific immune response pathways in pancreatic cyst fluid may serve as a biomarker of dysplasia.

An exploratory study carried out by Lee *et al.* utilised microarray assays to investigate the expression of inflammatory mediator proteins in pancreatic cyst fluid obtained from patients with pre-malignant BD-IPMNs or benign pseudocysts, which arose from a background of inflammation, as is typically the case [162]. Although this study was limited by sample size, the results showed differential inflammatory mediator protein profiles for pre-malignant and benign PCLs [162]. Eotaxin, hepatocyte growth factor (HGF), and granulocyte-macrophage colony-stimulating factor (GM-CSF) were expressed at lower concentrations in high-risk pre-malignant PCLs compared to in benign pseudocysts [162]. Eotaxin is a chemokine that promotes the selective recruitment of eosinophils, which are involved in the pathogenesis of several inflammatory disease such as eosinophilic oesophagitis, gastroenteritis, and pneumonia [163]. Similarly, in the pancreatic setting, murine models have demonstrated that the expression of GM-CSF plays a role in the recruitment of antigen-presenting immune cells, such as macrophages [164].

Additionally, MDSCs have been observed to populate human PCLs with high-grade dysplasia but are almost absent in lesions with low-grade dysplasia [165,166]. MDSCs can suppress the anti-tumour immune response through programmed cell death 1 receptor (PD-1) expression and release of reactive oxygen species [167,168]. Furthermore, MDSCs can also differentiate into anti-inflammatory M2 macrophages, which contribute to the immunosuppressive environment through the release of chemokines such as IL-10 and TGF-β [169]. This mounting data seems to suggest there is a dysregulated chemotactic mechanism in high-risk pre-malignant lesions, such as BD-IPMNs.

Prostaglandins play a critical role in the generation and resolution of inflammation. Differential expression levels of prostaglandin E_2_ (PGE_2_) in human pancreatic cyst fluid was observed between PCLs with high-grade dysplasia and invasive carcinoma [170]. Similarly, Yip-Schneider *et al.* found that expression levels of PGE_2_ in low-grade, high-grade, and invasive human IPMN cyst fluid increased in a stepwise manner [171]. PGE_2_ has been shown to influence the differentiation of human effector T cell populations such as Th1, Th2, Th17, Treg, T follicular helper cells, and circulating T lymphocytes [172]. Interestingly, this *in vitro* study highlighted the capacity of PGE_2_ to suppress Treg differentiation, particularly via the G-coupled protein receptor, EP2 [172]. This data contrasts previous findings of increased human Treg accumulation associated with the malignant progression of pre-neoplastic lesions [19,21]. With increased expression of both Treg cell subsets and PGE_2_ recorded in PCLs with high-grade dysplasia, perhaps dysregulation of EP2 receptor expression promotes Treg accumulation, permitting the immunosuppression in PCLs with higher risk of PC progression.

Interestingly, a number of studies have emerged demonstrating that mutant-specific KRAS inhibition can significantly transform the immune microenvironment of PC and PanIN lesions [[173], [174], [175]]. In a study by Mahadevan *et al.* using murine models, non-covalent small molecule KRAS inhibitors were shown to remodel the immune microenvironment of PanIN lesions and advanced PC from an “immunologically cold" state to an "immunologically hot" state, marked by increased immune cell infiltration and enhanced sensitivity to immunotherapies [175]. The effect of KRAS inhibition on the immune microenvironment of PCLs has not yet been elucidated. However, given that *KRAS* mutations are also identified in pre-malignant PCLs, KRAS inhibitors may be a promising avenue to investigate.

Distinct patterns of T cell responses have been shown to be associated with varying grades of dysplasia [165]. A pro-inflammatory immune response, comprised of cytotoxic T cells, activated T-helper cells, and dendritic cells, was readily observed in human PCLs with low-grade dysplasia [165]. In a study conducted by Enzler *et al*. where multiplex immunohistochemical staining was used to characterise the cellular immune component of surgically resected tissue from PCLs and PC, a high CD8^+^ *T* cell infiltration was identified in pre-malignant lesions [22,23]. Populations of T cells progressively decreased during progressive dysplasia in human PCLs, which suggests a diminished immune response or otherwise, a tumour escape mechanism during the process of tumour progression [165]. These findings were consistent with a study conducted by Maker *et al.* who investigated the expression of cytokines in human IPMN cyst fluid to determine anti-tumour immune response [75]. The results from this evaluation showed significantly higher concentrations of pro-inflammatory cytokines IL-1β, IL-5, and IL-8 in the presence of high-grade dysplasia or invasive carcinoma compared to low-risk PCLs with low-grade or moderate dysplasia [75]. Although IL-8 concentration was the most highly expressed among the assessed analytes and differentially expressed between low-risk and high-risk PCLs, when multivariate analysis was performed, only IL-1β remained a significant immunobiological biomarker of high-risk PCLs for the prediction of PC development [75].

There is an urgent unmet need for improved biological characterisation of pre-malignant PCLs to implicate appropriate management strategies for patients with high-risk pre-malignant PCLs. Previous studies have demonstrated the value of integrating immunobiological biomarkers into cancer staging, prognosis, and therapeutic decision-making [[147], [148], [149], [150], [151]]. As such, immunobiological parameters of pre-malignant PCLs, as in the case of immune cell infiltrates of CD8^+^ T cells, Tregs and MDSCs and/or the soluble immune mediators including IL-1β, IL-5 and IL-8, could be incorporated to further strengthen the accuracy of current PCL risk-stratification guidelines and inform therapeutic intervention strategies to prevent late-stage PC development (Table 2).

**Table 2:** Summary of immunobiological biomarkers under investigation in PCLs.

**Biomarker**	**Study type**	**Study Size**	**Key findings**	**Reference**
***Differences in immune cell profiles***				
CD8^+^*T* cellinfiltration	Single-cell transcriptomics of the stromal compartment of PC and precursor lesions	*n* = 6 (*n* = 2 PC, *n* = 2 LGD IPMN, *n* = 2 HGD IPMN)	CD8^+^*T* cell population increased in HGD-IPMN lesions vs LGD-IPMN lesions	[165]
	Spatial and phenotypic profiling of surgically resected tissue from PC and precursor lesions	*n* = 265 (*n* = 36 normal pancreatic tissue, *n* = 17 MCN, *n* = 71 IPMN, *n* = 29 PanIN, *n* = 8 IPMN-associated PDAC, *n* = 104 PDAC)	Increased CD8^+^*T* cell infiltration was observed in the microenvironment of MCN and even more so in IPMN lesions	[22,23]
Treg infiltration	Immunohistochemical analysis of surgically resected tissue from PC and precursor lesions	*n* = 264 (*n* = 15 non-neoplastic lesion, *n* = 51 IPMN, *n* = 198 PDAC)	CD4^+^ Treg frequency increased with increased dysplasia (non-neoplastic tissue from PDAC patients 7.9 %, non-neoplastic lesions 10.1 %, PDAC 34.6 %)	[19]
	Spatial and phenotypic profiling of surgically resected tissue from PC and precursor lesions	*n* = 214 (*n* = 21 normal pancreatic tissue, *n* = 16 MCN, *n* = 49 IPMN, *n* = 20 PanIN, *n* = 8 IPMN-associated PDAC, *n* = 100 PDAC)	Infiltration of Tregs was low in the microenvironment of cystic lesions MCN and IPMN and elevated in IPMN-associated PDAC	[22,23]
MDSC infiltration	Single-cell transcriptomics of the stromal compartment of PC and precursor lesions	*n* = 6 (*n* = 2 PC, *n* = 2 LGD IPMN, *n* = 2 HGD IPMN)	MDSC population enrichment in IPMN lesions with high-grade dysplasia (3.5 %) vs IPMN lesions with low-grade dysplasia (2.3 %)	[165]
***Differences in immune factor expression***				
Eotaxin, HGF, GM-CSF	Multiplex inflammatory mediator protein-targeted microarray	*n* = 10 (*n* = 5 BD-IPMN, *n* = 5 inflammatory cysts)	Concentration of eotaxin, HGF, and GM-CSF was significantly decreased in BD-IPMNs vs. inflammatory cysts	[162]
PGE_2_	PGE_2_ concentration assessed by ELISA in pancreatic cyst fluid	*n* = 101 (*n* = 47 LGD/moderate dysplasia IPMN, *n* = 34 HGD IPMN, *n* = 20 invasive IPMN)	Concentration of PGE_2_ increased with increased dysplasia (LGD-IPMN 1.2 pg/µL, HGD-IPMN 3.5 pg/µL, IPMN with invasive carcinoma 4.4 pg/µL	[171]
IL-1β, IL-5, IL-8	Cytokine concentration assessed by multiplex ELISA in pancreatic cyst fluid	*n* = 40 (*n* = 21 low-risk cysts defined by low-grade or moderate dysplasia, *n* = 19 high-risk cysts defined by high-grade dysplasia or invasive PC)	Concentration of pro-inflammatory cytokines was significantly increased in high-risk cysts vs. low-risk cysts (IL-1β: 539 pg/µL vs. 0.2 pg/µL, IL-5: 0.2 pg/µL vs. 0.05 pg/µL, IL-8: 8089 pg/µL vs. 2758 pg/µL).	[75]

LGD=low-grade dysplasia. HGD=high-grade dysplasia. IPMN=intraductal papillary mucinous neoplasm. MCN=mucinous cystic neoplasm. PC=pancreatic cancer. PDAC=pancreatic ductal adenocarcinoma. MDSC=myeloid-derived suppressor cell. HGF=hepatocyte growth factor. GM-CSF=granulocyte-macrophage colony-stimulating factor. BD-IPMN=branch duct-IPMN. PGE_2_=prostaglandin E2. ELISA=enzyme-linked immunosorbent assay.

## Conclusions

Given the 5-year survival rate of PC reaching only 13 %, in conjunction with the vast majority of late-stage clinical disease and resistance to mainstay therapies, this highlights the crucial need for earlier diagnosis and intervention of PC. At present, a number of recognised risk-factors are associated with the biological establishment of PC, one of which includes pre-malignant PCLs. Some PCLs possess the ability to undergo malignant transformation and are regarded as precursor lesions of PC and represent a unique opportunity for early disease interception and intervention. However, there is a notable geographical and institutional lack of consensus on current risk-stratification guidelines for the classification and management of pre-malignant PCLs. Further refining the knowledge of crosstalk between the immune landscape and pancreatic microenvironment across different PCL subtypes is crucial to develop more in-depth and well-informed risk-stratification guidelines for the management of PCLs. Incorporating differential immunobiological features of pre-malignant PCLs, such as immune cell infiltration and cytokine profiles, into existing risk-stratification and management guidelines may potentially provide a more nuanced assessment of malignant potential. This immunobiological-informed approach offers potential to transform current management strategies by identifying high-risk lesions earlier, guiding surveillance intensity, and ultimately reducing the incidence of late-stage PC diagnoses.

## CRediT authorship contribution statement

**Rebecca Lyons:** Writing – review & editing, Writing – original draft, Conceptualization. **Stephen G. Maher:** Writing – review & editing, Conceptualization. **Joanne Lysaght:** Writing – review & editing, Conceptualization.

## Declaration of competing interest

The authors declare that they have no known competing financial interests or personal relationships that could have appeared to influence the work reported in this paper.
